# Carbon sequestration potential of plantation forests in New Zealand - no single tree species is universally best

**DOI:** 10.1186/s13021-024-00257-1

**Published:** 2024-04-05

**Authors:** Serajis Salekin, Yvette L. Dickinson, Mark Bloomberg, Dean F. Meason

**Affiliations:** 1 Scion Research Ltd. (New Zealand Forest Research Institute), Rotorua, 3046 New Zealand; 2 New Zealand School of Forestry, University of Canterbury, Christchurch, 8041 New Zealand

**Keywords:** Carbon forestry, Plantation forest, Carbon sequestration, Site-species matching, Forest carbon

## Abstract

**Background:**

Plantation forests are a nature-based solution to sequester atmospheric carbon and, therefore, mitigate anthropogenic climate change. The choice of tree species for afforestation is subject to debate within New Zealand. Two key issues are whether to use (1) exotic plantation species versus indigenous forest species and (2) fast growing short-rotation species versus slower growing species. In addition, there is a lack of scientific knowledge about the carbon sequestration capabilities of different plantation tree species, which hinders the choice of species for optimal carbon sequestration. We contribute to this discussion by simulating carbon sequestration of five plantation forest species, *Pinus radiata*, *Pseudotsuga menziesii*, *Eucalyptus fastigata*, *Sequoia sempervirens* and *Podocarpus totara*, across three sites and two silvicultural regimes by using the 3-PG an ecophysiological model.

**Results:**

The model simulations showed that carbon sequestration potential varies among the species, sites and silvicultural regimes. Indigenous *Podocarpus totara* or exotic *Sequoia sempervirens* can provide plausible options for long-term carbon sequestration. In contrast, short term rapid carbon sequestration can be obtained by planting exotic *Pinus radiata, Pseudotsuga menziesii* and *Eucalyptus fastigata*.

**Conclusion:**

No single species was universally better at sequestering carbon on all sites we tested. In general, the results of this study suggest a robust framework for ranking and testing candidate afforestation species with regard to carbon sequestration potential at a given site. Hence, this study could help towards more efficient decision-making for carbon forestry.

**Supplementary Information:**

The online version contains supplementary material available at 10.1186/s13021-024-00257-1.

## Background

Anthropogenic greenhouse gas emissions accelerate climate change with widespread negative impacts on ecosystems and society [1]. While long-term solutions for reducing and mitigating the emission of greenhouse gases are vitally important, urgent short-term actions are also needed to meet national goals for emissions reduction [2]. Afforestation (planting new forests on previously unforested land) can sequester atmospheric carbon in the short to medium term [3, 4]. As such, it can be a tool to mitigate anthropogenic climate change.

### The New Zealand emissions trading scheme

New Zealand’s Emissions Trading Scheme (NZ ETS) was designed to reduce greenhouse gas emissions and assist the New Zealand Government in meeting international obligations set for 2050 [5]. The NZ ETS requires businesses to measure and report their greenhouse gas (GHG) emissions and surrender one emissions unit (an NZU) for each emitted tonne of carbon dioxide equivalent (t CO_2_e). Conversely, businesses that reduce GHG in the atmosphere will receive NZUs. Also, businesses participating in the NZ ETS can buy and sell units from each other, with NZU prices reflecting supply and demand within the ETS.

Afforestation can act as a carbon sink by sequestrating and storing more CO_2_ than it releases, effectively offsetting GHG emissions. Therefore, owners of Kyoto-compliant plantation forests (established onto pasture or other low-stature vegetation since 1 January 1990) can join the NZ ETS and receive NZUs for CO_2_ sequestered by their forests [6]. Forest owners can then sell their NZUs and receive income from the sale.

Options for afforestation within the NZ ETS are subject to debate within New Zealand. Two key issues are whether to use (1) exotic plantation species versus indigenous forest species and (2) fast growing short-rotation species versus slower growing species that can be grown over long rotations of 50 years or more [7–9]. These debates are constrained by the lack of robust information on the amount and rates of carbon sequestered by a full range of candidate tree species, across the range of available afforestation sites [10, 11]. It is difficult to rigorously compare forestry options when information on their growth and carbon sequestration rates is limited or has been derived using disparate methods, many of which are not publicly accessible. Therefore, there is potential to offer improved information to support decision-makers by predicting species- and site-specific carbon sequestration rates over time.

### Modelling forest productivity and carbon sequestration

Globally, many studies have investigated the productivity and potential of species’ carbon sequestration rates under plantation forests [4, 12, 13]. In New Zealand, Hall [14] estimated the long-term carbon sequestration of an indigenous forest and a *Pinus radiata* (D. Don) stand transitioning to an indigenous forest at a site in the South Island of New Zealand. Other indigenous tree species where productivity and/or carbon sequestration have been studied include mānuka (*Leptospermum scoparium* J.R.Forst. & G.Forst. and kānuka (*Kunzea ericoides*(A.Rich.) Joy Thomps.) [15], mountain beech (*Fuscospora cliffortioides* (Hook.f.) Heenan & Smissen) [16], mixed species shrublands [17], shrubs and post-1989 natural forests [18], planted indigenous forests [19], young indigenous plantations [20], tōtara *(Podocarpus totara* G. Benn. Ex D. Don) and kauri (*Agathis australis* (D. Don) Lindl) [21–23]. Similarly, major exotic plantation forest tree species have been modelled and compared over time. In addition to *P. radiata* and *Pseudotsuga menziesii* (Mirbel) Franco, these studies included several *Eucalyptus* species and *Sequoia sempervirens* (D. Don) Endl [10, 24–29].

Forest productivity varies spatially and temporally in a complex way, driven by site conditions interacting with tree genotypes and silviculture [30, 31]. Modelling is a robust way to describe and quantify these complexities [32]. The main essence of the model is not to reproduce every detail of any biological system; rather, it should be an optimised balance between exclusion and retention, simplicity and complexity. It is particularly important for plantation forestry, where a broad range of sites and silvicultural options can be simultaneously tested to make informed decisions. Selecting a statistically precise model can lead to a “forecast trap”, where the model makes accurate predictions within its domain but does not address the possibility of a better outcome, which may occur outside of the model’s domain [33]. The implication is that a single highly precise model might be adequate to simulate a certain scenario, but a broader set of less-correct models may be more useful for good decision-making from poorly understood processes and accommodate wider variations in scenarios.

Empirical, statistical/parametric and nonparametric models have proven accurate in predicting forest growth and yield and are simple yet robust [34]. However, these models cannot provide any ecophysiological understanding and do not describe processes leading to differing growth and yield within and between sites. Despite their complexity, ecophysiological models provide that information [35]. Few studies have focused on the ecophysiological aspects of carbon forestry, which is establishing plantation forests to ensure maximum carbon sequestration with the goal of mitigating climate change [36–38]. However, a clear scientific gap exists to improve predictions of carbon sequestration rates based on species, genetics, site characteristics, and silvicultural treatments.

Only a handful of publications have compared the productivity of various tree species and the influence that genetics, environment, silviculture, and their interactions have on DBH, height, timber volume or biomass growth [e.g. 39]. An ecophysiological model, such as “Physiological Principles in Predicting Growth” (3-PG) [36, 40–42] can meet this need to describe and explain forest growth in terms of genetics, environment and silviculture. 3-PG is a robust, widely accepted model that can provide information about underlying processes with appropriate biomass partitioning [43] and can be extended to model novel conditions beyond the parametrisation data. It has been parametrised and used for a range of species and sites globally, providing robust projections of forest growth [for example, 44, 45–47].

### Research aim

This study aims to provide a framework to enable accurate and realistic comparisons of carbon sequestration rates amongst candidate afforestation species. This comparison can inform current carbon forestry debates and support policy and forest management decisions within New Zealand. It can also demonstrate a methodology to resolve similar debates internationally. To provide the data necessary, an ecophysiological model is needed. 3-PG was used to quantify and compare the likely carbon sequestration of five plantation forest species: *P*. *radiata*, *Ps. menziesii*, *E. fastigata*, *S. sempervirens* and *P. totara* across three sites with differing site characteristics and two silvicultural regimes (three regimes for *P. radiata*).

## Methods

### Description of scenarios

New Zealand has complex topography, climate and geography. Its mid-latitude location astride the circumpolar westerly wind belt produces considerable regional differences in weather and climate that are reinforced by the effects of an axial mountain chain extending the length of the country from northeast to southwest. The interaction of the prevailing westerly winds and the NE-SW mountain chain produces a sharper climatic contrast from west to east than from north to south [48].

**Fig. 1:**
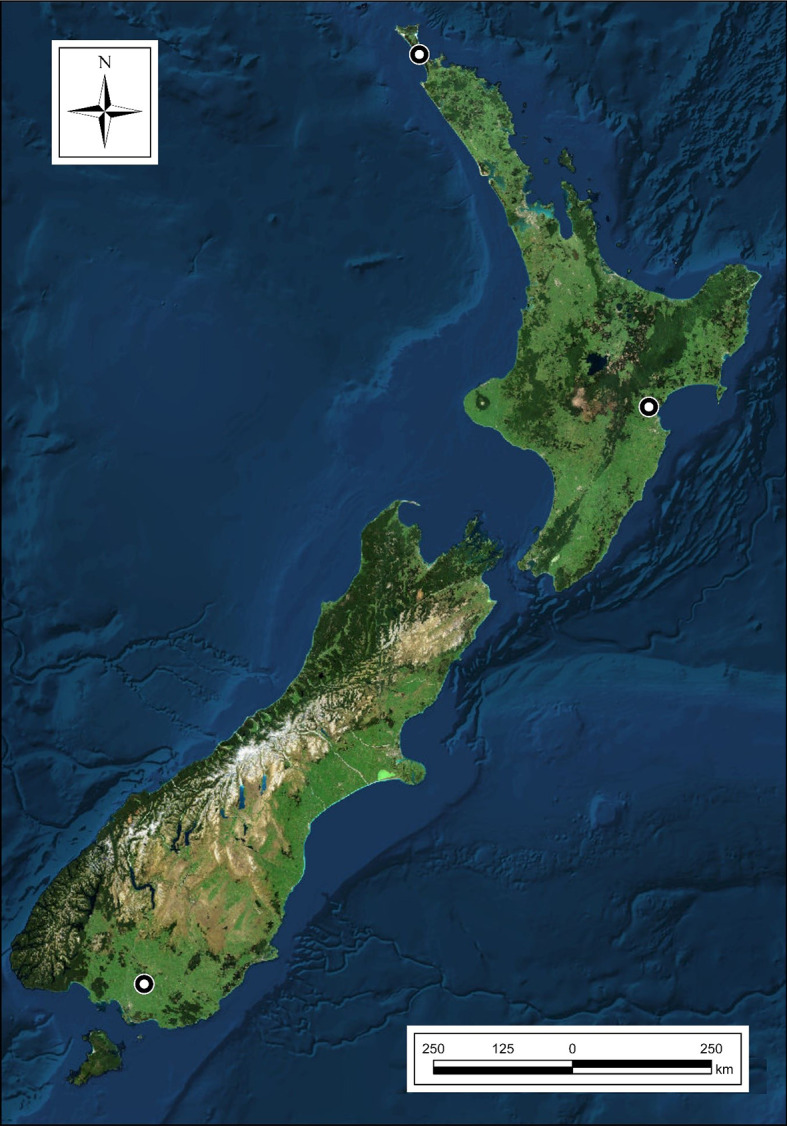
Experimental locations

The most recent classification of climate regions for the country was undertaken by Garr and Fitzharris [49], resulting in 18 regions differentiated by temperature and precipitation. Our study sites’ locations were chosen to (1) reflect the range of these climate regions and (2) the range of sites suitable for plantation forests. The location options were narrowed by discarding all forest reserves, national parks, water bodies and other areas unsuitable or unavailable for afforestation. Then we chose three locations, one each in the Central North Island (CNI), Northland (NL) and Southland (SL), respectively. Contrasting edaphic and climatic characteristics, historically documented productivity for plantation forests and overall coverage of New Zealand’s land area were considered for site selection. The CNI has a high proportion of the most productive forestry sites across New Zealand due to mild temperatures (mean annual temperature of 13.8 °C) and plentiful well-distributed rainfall. The NL location represents the warmest climate in New Zealand, with mean annual temperatures of 15 °C in coastal areas. The SL location is in the coolest lowland region in New Zealand, with mean annual temperatures of 9.8 °C and low sunshine hours [49]. Edaphic and climatic characteristics of these sites are provided in Table 1, and locations are shown in Fig. 1.

**Table 1:** Site descriptions. Standard deviations are shown in brackets

Site	Location		Soil		Elev. (m asl)	Climate				
	Lat. (°)	Long. (°)	Soil Texture	ASW (mm)		Temperature (°C)		Precipitation (mm/annum)	Mean radiation (MJ m^− 2^day^− 1^)	Frost (days/annum)
						Mean maximum	Mean minimum			
Central North Island (CNI)	−39.341	176.752	Sandy loam	250	363	17.80 (± 4.47)	8.76 (± 3.5)	1350 (± 21.8)	14.2 (± 6.41)	17 (± 3.66)
Southland (SL)	−46.1735	168.595	Clay loam	250	680	15.00 (± 4.41)	5.18 (± 3.0)	900 (± 30.8)	12.5 (± 7.43)	46 (± 7.92)
Northland (NL)	−34.8008	173.0418	Sandy loam	150	63	19.80 (± 3.43)	12.70 (± 2.8)	1700 (± 21.8)	14.8 (6.12)	0

We chose to study the five selected species because of their current or potential use in New Zealand’s plantation forests and the availability of adequate information to parametrise the 3-PG model [22, 28, 50, 51]. Two target silvicultural scenarios, the first one aimed at wood production (Production) and the second one intended for carbon sequestration (Carbon), were modelled for all species except for *Pinus radiata*, where the Production scenario was also run with pruned and unpruned options to reflect business-as-usual plantation management in New Zealand. For the Carbon scenario, a relatively high planting density (stems ha^− 1^) and no silvicultural interventions after establishment were chosen. On the other hand, the Production scenario had lower planting density for some species (e.g., *E. fastigata*) and regular/planned silvicultural interventions, i.e., thinning and pruning. Detailed silvicultural scenarios are shown in Table 2.

**Table 2:** Species-specific description of different target silvicultural scenarios

Species	Simulation period (years)	Target scenario	Planting density (stems/ha)	Thinning		Pruning ages (years)	Reference
				Age (years)	Stems retained (stems/ha)		
*Pinus radiata*	60	Production	1000	10	600	-	Moore et al. [89]; Watt et al. [90]
	60	Production (pruned)	1000	8	350	5, 6 & 8	Paul et al. [91]
	60	Carbon	1000	-	-	-	
*Eucalyptus fastigata*	50	Production	800	8	200	8	Paul [91], Nicholas [92]
	50	Carbon	1200	-	-	-	
*Pseudotsuga menziesii*	70	Production	1400	15	500	-	Paul [91], Maclaren [93]
	70	Carbon	1500	-	-	-	
*Podocarpus totara*	90	Production	1750	20	1000	-	Bergin and Kimberley [23]; Paul [91]
				40	500		
				50	300		
	90	Carbon	1750	-	-	-	
*Sequoia sempervirens*	90	Production	900	11	300	4, 6 & 9	Paul [91], Meason et al. [94]
	90	Carbon	900	-	-	-	

### Data

The edaphic and climatic data for the three study sites were extracted from two sources. The first source was the National Institute of Water and Atmospheric Research (NIWA), which operates meteorological stations throughout New Zealand. These measurements are interpolated daily for the whole country on a regular (∼ 5 km) grid called the virtual climatic station network (VCSN) [52]. A 30-year normal average dataset was available for this study. Each of our areas was assigned to its nearest VCSN point to extract monthly maximum and minimum temperature (°C), total monthly precipitation (mm), total monthly radiation (MJ m^− 2^), and the number of frost days per month (Table 1).

The second data source was the Fundamental Soil Layer (FSL) geodatabase which describes soil physical and chemical characteristics throughout New Zealand [53]. In FSL, only the soil unit within each map polygon was identified in the field, with individual soil characteristics then correlated from the soil unit [53]. The required soil characteristics were extracted using the FSL layer through ArcMap 10.8 (ESRI, Redlands, CA) (Table 1). Extracted soil characteristics included soil texture and available soil water (ASW, mm).

### Model simulation and carbon prediction

All target scenarios were run in 3-PG to predict and compare their rates and total amounts of above-ground carbon sequestration. The 3-PG model is a generalised ecophysiological (process-based) tree growth model. It has been used widely around the globe for many monospecific and mixed-species stands [e.g., 42, 54, 55, 56]. 3-PG is a stand-level model, first developed by Landsberg and Waring [41], that requires a combination of abiotic and biotic variables as inputs to simulate observed growth and forecast future changes to tree biomass and productivity [57, 58]. The 3-PG model and its variants use subroutines to predict net primary productivity (NPP), transpiration, respiration, and growth. Absorbed photosynthetically active radiation (APAR) is calculated as a function of photosynthetically active radiation (PAR) and leaf area index (LAI). The utilised portion of APAR (APARu) is calculated using a set of dimensionless modifiers with values varying from zero (total constraint to utilisation) to 1 (no constraint to utilisation). Suboptimal and supraoptimal temperatures, high vapour pressure deficits (VPD), infertile soils, and deficits in available soil water (ASW) combine to constrain the utilisation of APAR and affect the growth and allocation of dry mass [29].

We employed the 3-PGjs variant of the 3-PG model [v 2.7; 59] to simulate the silvicultural scenarios (Table 2) for the three different sites (Table 1). Over time, the 3-PGjs variant has been parameterised, calibrated and validated for all the studied species with the site and species-specific data in New Zealand [25, 28, 29, 50, 51]. While we do not intend to explain all the calibration and validation procedures in this study, we do provide all species-specific parameters and their definition as supplementary materials (Table S1). As it is difficult to appropriately quantify species- and site-specific soil fertility modifiers, static species-specific soil fertility modifiers were used following earlier calibration studies.

We simulated partitioned foliage (including branch) and stem oven dry biomass (ODW, t ha^− 1^) for the different silvicultural scenarios from age two years to a defined species-specific simulation period. Then, the ODW of each biomass component was converted to its equivalent weight of carbon using species-specific carbon fractions (Table 3). Finally, each biomass component was combined to represent the total above-ground sequestered carbon according to the IPCC [60]. Furthermore, Oliver et al. [61] confirmed that simulated *E. fastigata* biomass partitioning from 3-PG closely followed measured biomass components.

**Table 3:** Species-specific carbon fractions (* IPCC default, species-specific information unavailable)

Species	Carbon fractions (CF) for each biomass component		Reference
	Foliage	Stem	
*P. radiata*	0.51	0.50	[95]
*E. fastigata*	0.51	0.51	[96]
*Ps. menziesii*	0.531	0.488	[97]
P. totara*	0.50	0.50	[60]
*S. sempervirens*	0.495	0.53	[37, 98]

### Statistical analysis

For the carbon-only scenario, the mean annual increment of above-ground carbon sequestration (MAIc, tC ha^− 1^year^− 1^) was calculated for short (25 years) and long (50 years) rotations for all investigated tree species. Each species was ranked from highest to lowest carbon sequestration MAIc at different sites and rotation lengths. Then, we compared (i) site-specific MAIc per species (over both rotation lengths) and (ii) rotation-specific MAIc per species (over all sites) to test significant differences between species ranks across sites and rotation lengths. We statistically tested these comparisons using a nonparametric pseudo-rank test for multiple contrast test procedure (MCTP) [62, 63]. This procedure allows for testing an arbitrary purely nonparametric multiple linear hypothesis without assuming homogeneous variances of the data, and the computation is compatible with simultaneous confidence interval (SCI); in particular, the distributions can have different shapes even under a null hypothesis [62]. The “rankFD” package applied the rank-based tests at a 95% confidence interval with normal approximation [63]. Data organisation and plotting were carried out in the R statistical environment through the “tidyverse” package [64–66].

## Results

### Above ground carbon sequestration

**Fig. 2:**
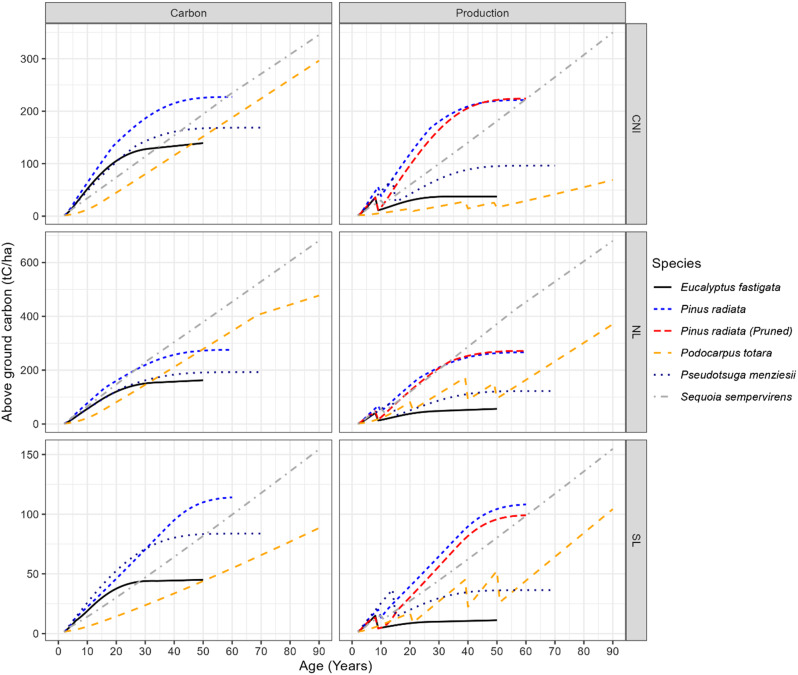
Site- and Species-specific above-ground carbon (tC ha^− 1^). CNI = Central North Island, NL = Northland and SL = Southland. Carbon and Production regimes are described in Table 2

**Fig. 3:**
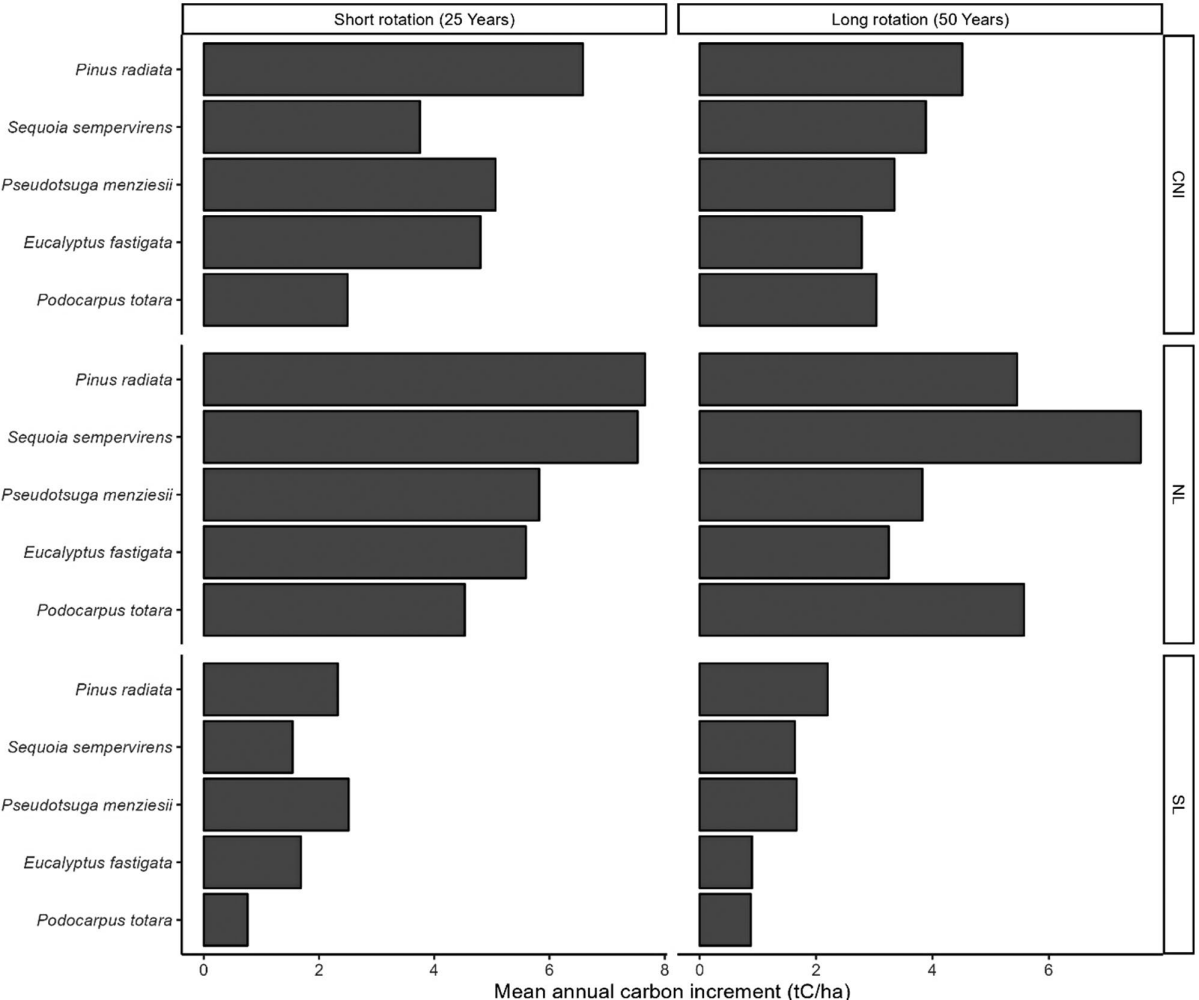
Species- and site-specific MAIc for short and long rotation (Carbon scenarios). Refer to Table 1; Fig. 1 for site details

Over all species, rotation lengths, and silvicultural scenarios, the Northland (NL) site sequestered the highest quantity of above-ground carbon (tC ha^− 1^) (Figs. 2 and 3). Species-wise, *P. radiata* had the highest above-ground carbon sequestration for the first 30 years, with one exception (*Ps. menziesii* in the SL site was marginally higher until age 30 years, then declined to a lower value than *P.radiata*). However, for periods longer than 30 years, the species with the highest carbon sequestration varied by site, silvicultural regime and simulation period, as described below.

Carbon sequestration of the Carbon and Production scenarios did not markedly vary for *P.radiata* and *S. sempervirens*. Carbon sequestration for pruned and unpruned *P. radiata* Production scenarios converged after age 30 years, except for the Southland site, where pruned *P. radiata* had less carbon (difference of 13.5 tC ha^− 1^) at the end of the simulation (Fig. 2).

In contrast, the thinning applied to the Production scenarios for *Ps. menziesii*, *P. totara* and *E. fastigata* resulted in markedly less carbon sequestration than the Carbon scenarios. In all cases, *P. totara* was the slow starter; therefore, it sequestered the lowest amount of carbon (tC ha^− 1^) at an early age (5–20 years). This changed from 20 years onward with the *P. totara* sequestration rate almost constant throughout the defined simulation period with no decline even after 90 years. Conversely, *E. fastigata* showed rapid initial carbon sequestration, especially for the Carbon scenarios, but reached an asymptote after 30 years.

### MAI of carbon sequestration

Site- and rotation-specific rankings according to above-ground MAIc for different species were significantly different using the nonparametric multiple contrast test procedure (MCTP) (Table 4). The detailed ranking summary and descriptive MCTP statistics are provided in the supplementary information (Table S2). For Carbon scenarios, short-rotation MAIc (tC ha^− 1^yr^− 1^) was led by *P. radiata*, with values ranging from 2.1 to 7.7, except SL where *Ps. menziesii* (2.5) had the highest MAIc. On the other hand, the best long-rotation MAIc for carbon-only scenarios varied with site and species. CNI and SL were led by *P. radiata* with values of 4.5 and 2.2, respectively, whereas NL was led by *S. sempervirens* (7.6) and followed by *P. totara* (5.5) and *P. radiata* (5.3), respectively.

**Table 4:** Summary statistics of nonparametric rank based MCTP (ANOVA type statistic)

Model	Rank ∼ Species × Rotation		Rank ∼ Species × Site	
Variables	Statistic	p-value	Statistic	p-value
Species	72.79	0.0007	124.25	0.0001
Rotation	35.03	0.0043	-	-
Species × Rotation	12.74	0.0120	-	-
Site	-	-	0.49	0.6300
Species × Site	-	-	10.7	0.0100

## Discussion

### Effect of species choice and site

Species rankings for MAIc were significantly different, suggesting that some species were consistently superior to others across all sites, rotations and silvicultural scenarios.

Overall, *P. radiata* performed well and showed sustained growth over the simulated period. Woollons and Manley [67] analysed growth data for *P. radiata* beyond the normal 25- to 30-year commercial rotation in New Zealand and confirmed its capacity for sustained growth for 60 years or more. *P. radiata* can tolerate a broad range of sites and climates [68] and has undergone several decades of tree improvement to ensure optimum gain [69]. Another common New Zealand wood production species, *S. sempervirens* is reported to have higher growth on some sites and often surpasses *P. radiata* growth at ages above 30 years, especially at North Island sites (i.e., CNI and NL) [70].

Besides *P. radiata* and *S. sempervirens*, *Ps. menziesii* showed high carbon sequestration, especially under the unthinned Carbon scenarios and for the SL site. The overall growth potential of *Ps. menziesii* is higher in New Zealand than elsewhere in the world, especially compared with its native origin, western North America. Waring et al. [71] explained this extraordinary growth rate through milder environmental stress, including temperature, solar irradiance, air humidity deficits and frost frequency in New Zealand compared with western Oregon, USA. However, the ascomycete fungus *Phaeocryptopus gaeumannii* (T. Rhode) Petr. occurs naturally as a microparasite within needles of *Ps. menziesii*, reported causing significant volume growth reduction in New Zealand, i.e. 35% in the North Island and 23% in the South Island, respectively [72]. *P. gaeumannii* is modulated by climatic factors, mean daily winter temperature and spring moisture [73]. Therefore, combining such biotic with abiotic stresses into a growth modelling framework is necessary [74, 75], as similar pathogenic stresses are expected to affect other plant species with future climate change [76]. This study did not consider the impacts of pathogens such as *Phaeocryptopus gaeumannii*.

Site effects interacting with species did have a significant effect on species rankings. For example, over the long simulation period *S. sempervirens* outperformed *P. radiata* for the NL site, was similar for the CNI site, but underperformed compared with *P. radiata* for the SL site. Species-specific temperature effects may largely drive this. In this study, precipitation was unlikely to limit tree growth for all three sites, and the ASW for the clay loam on the SL site was superior to that for the sandy loam on the NL site. The NL site (elevation 63 m, mean maximum and mean minimum temperatures 19.8 and 12.7 °C and zero frost days) is more favourable to growth than the SL site (elevation 680 m, mean maximum and mean minimum temperatures 15.0 and 5.18 °C and 46 frost days). The higher growth performance of forests in NL and CNI also stands out in other growth modelling studies for plantation forests in New Zealand [e.g., 10, 26], and temperature is identified as a strong driver of tree productivity in these studies.

*Sequoia sempervirens* and *P. totara* are long-lived conifers [77, 78]. Unlike the other three species, they showed linear increases in biomass and sequestrated carbon to the end of their simulation period (90 years) rather than a sigmoid growth form with an asymptote (maximum attainable biomass or yield per unit area) [79, 80] shown by the other three species. Pretzsch [81] reported that the stand growth asymptote can be attained beyond the usual rotation age and is influenced by management and climatic factors. It appears that *S. sempervirens* and *P. totara* may be capable of linear and sustained growth for longer periods than the other studied species.

### Effect of species choice and silviculture

The two rotation lengths were significantly different regarding the rank of their MAIc. The MAIc for the longer (50-year) rotation was less than for the 25-year rotation for most species. The interaction of rotation length with species was also significant, largely driven by the improvement in rank for *P.totara* and *S. sempervirens*. In the long term, MAIc for *S. sempervirens* and *P. totara* gradually surpassed *P. radiata* and *Ps. menziesii*, and *S. sempervirens* had a superior ranking to *Ps. menziesii* for the 50-year rotation in NL and CNI. Sensitivity to edaphoclimatic factors at the establishment phase of *S. sempervirens* may play a crucial role in its slow initial sequestration [82]. The initial carbon sequestration rate of *P. totara* can be explained by its shade-tolerant nature and initial competition with other vegetation, significantly reducing early growth [22, 23]. *P. radiata* rankings were lower for long versus short rotations, whereas the reverse applied for *P. totara*.

While the silvicultural regime did not markedly influence the carbon sequestration of *P.radiata* and *S. sempervirens*, it had a larger influence on the outcomes of the *Ps. menziesii*, *P. totara* and *E. fastigata* scenarios. Oliver and Larson [83] note that after thinning or other stand disturbance, how rapidly the residual crop trees respond depends on the crown and root expansion rates. These expansions of crown and root systems depend on a combination of stand and site characteristics— crown and root characteristics, tree age, site characteristics, tree vigour and amount of growing space released by the thinning. Ultimately, the synecology of each species (e.g. shade-tolerance, competitive ability, growth rate and allometry) dictates the specific impacts of various silvicultural choices [83]. The results of our study suggest that, in the Production scenarios, *P.radiata* and *S. sempervirens* responded more rapidly to thinning than the other three species.

### Limitations of the modelling approach

It is to be noted that any modelling simulation is a simplification of the present to project the future [84]. Consequently, capturing every factor affecting the model is impossible; some less understood and unincluded factors may substantially affect growth [85]. In addition, future climate change will potentially affect forest tree growth and therefore carbon sequestration [86]. Climate-induced biotic and abiotic disturbances and natural regeneration were not included during the simulation, but these can collectively affect carbon sequestration [87, 88]. Therefore, it will be prudent to include simulations with different climate change scenarios. Using a physiological process-based model, such as 3-PG, provides the opportunity to simulate a range of potential climate change scenarios and provides an understanding of the likely impact of climate change on carbon sequestration of forests.

While the forest model used in this study has been adequately parametrised and validated, work is needed to further evaluate model predictions against the observed growth of plantation forest species in New Zealand. Model predictions depend on the parameter assumptions used in the initial calibration process and require further testing. Other areas for improvement include (1) allometric analyses to estimate carbon sequestered in different tree components, (2) for the Production scenarios, additional life-cycle analysis of the harvested wood products and (3) modelling the effect of abiotic and biotic stressors such as pests and diseases, frosts and drought (e.g., the impact of *P. gaeumannii* on *Ps. menziesii*, as discussed above). Furthermore, because indigenous tree species are represented in this study only by *P. totara*, it will be useful to expand the model to other candidate indigenous species for afforestation., e.g., southern beech (*Fuscospora* spp).

## Conclusions

This study is one of the few that systematically makes site-, regime- and species-specific comparisons of carbon sequestration by plantation forests. The results allow us to directly compare the likely carbon sequestration of the studied species. The model results from these sites are not used to draw general conclusions about carbon sequestration of forests in the broader regions. Further, the results of this study show that while tree species can achieve rapid carbon sequestration, site-species matching must be practised appropriately, as no single species is universally better at sequestering carbon on all sites. The key is to match the species silvics and silviculture to the site to ensure sustainable long-term carbon sequestration.

But perhaps more important than the specific results at the three sites studied, this study demonstrates that process-based models such as 3-PG may be used to compare the carbon sequestration of various species at sites of interest. Furthermore, the variation in carbon sequestration among the scenarios suggests that forest owners and investors interested in sequestering carbon should use such tools, parameterised to fit their context, to inform their decisions and select species, sites and silvicultural regimes.

Finally, using a process-based model may provide an opportunity to investigate the likely impact of climate change on carbon sequestration to be investigated. Climate change is likely to be already impacting the growth and carbon sequestration of forests in various ways, including changes to the amount and distribution of precipitation, and humidity and temperature regimes. However, climate change is a complex global phenomenon, and it is difficult (or impossible) to empirically provide future projections of impacts. These impacts are likely to become more extreme over time, and it would be wise to consider the influence of climate change on the sequestration and retention of carbon if we intend to mitigate climate change. Future research directions should investigate how a range of climate change scenarios influence the likely carbon sequestration of these candidate tree species using process-based models and whether the preferred species, sites, and silvicultural regimes change when climate change impacts are considered.

### Electronic supplementary material

Below is the link to the electronic supplementary material.

Supplementary Material 1

## Data Availability

All the data and the model (3-PG) used are publicly available and no additional data collected or generated during this study.

## Abbreviations

NZ ETS: New Zealand’s Emissions Trading Scheme
GHG: Green House Gasses
NZU: New Zealand Unit
CNI: Central North Island
NL: Northland
SL: Southland
VCSN: Virtual Climatic Station Network
FSL: Fundamental Soil Layer
ASW: Available Soil Water
NPP: Net Primary Productivity
APAR/u: Absorbed Photosynthetically Active Radiation / utilised portion
PAR: Photosynthetically Active Radiation
LAI: Leaf Area Index
ODW: Oven Dry Weight (Biomass)
MAIc: Mean Annual Increment of above-ground carbon sequestration
MCTP: Multiple Contrast Test Procedure
SCI: Simultaneous Confidence Interval
DBH: Diameter at Brest height (at 1.4 m for New Zealand)
