# Supplementary material for: Carbon sequestration potential of plantation forests in New Zealand - no single tree species is universally best
Source: Carbon Balance Manag. 2024 Apr 5;19:11. doi: 10.1186/s13021-024-00257-1 (PMC10998325; doi:10.1186/s13021-024-00257-1)
Supplement: Supplementary file 1 — Supplementary Material 1 [file 13021_2024_257_MOESM1_ESM.docx]

**Appendix**

**Table S1. Species-specific parameters for 3-PG model simulation.**

| **Meaning/comments** | **Name** | **Units** | ***P. radiata*** | ***E. fastigata*** | ***S. sempervirens*** | ***Ps. menziesii*** | ***P. totara*** |
| --- | --- | --- | --- | --- | --- | --- | --- |
| ***Biomass partitioning and turnover*** |  |  |  |  |  |  |  |
| *Allometric relationships & partitioning* |  |  |  |  |  |  |  |
| Foliage:stem partitioning ratio @ D=2 cm | pFS2 | - | 0.80 | 1.00 | 0.80 | 1.00 | 0.81 |
| Foliage:stem partitioning ratio @ D=20 cm | pFS20 | - | 0.40 | 0.10 | 0.40 | 0.15 | 0.22 |
| Constant in the stem mass v. diam. relationship | aS | - | 0.0095 | 0.158 | 0.0243 | 0.0062 | 0.0455 |
| Power in the stem mass v. diam. relationship | nS | - | 2.72 | 2.10 | 2.72 | 3.10 | 2.5243 |
| Maximum fraction of NPP to roots | pRx | - | 0.60 | 0.85 | 0.60 | 0.80 | 0.60 |
| Minimum fraction of NPP to roots | pRn | - | 0.25 | 0.30 | 0.25 | 0.25 | 0.25 |
| *Litterfall & root turnover* |  |  |  |  |  |  |  |
| Maximum litterfall rate | gammaFx | 1/month | 0.0300 | 0.0270 | 0.0500 | 0.0275 | 0.0300 |
| Litterfall rate at t = 0 | gammaF0 | 1/month | 0.0010 | 0.0035 | 0.0010 | 0.0010 | 0.0010 |
| Age at which litterfall rate has median value | tgammaF | months | 36 | 12 | 36 | 12 | 36 |
| Average monthly root turnover rate | gammaR | 1/month | 0.015 | 0.015 | 0.015 | 0.015 | 0.015 |

| **Meaning/comments** | | **Name** | **Units** | | ***P. radiata*** | ***E. fastigata*** | ***S. sempervirens*** | | ***Ps. menziesii*** | ***P. totara*** |
| --- | --- | --- | --- | --- | --- | --- | --- | --- | --- | --- |
| **NPP & conductance modifiers** |  | | |  |  |  | |  |  |  |
| *Temperature modifier (fT)* |  | | |  |  |  | |  |  |  |
| Minimum temperature for growth | Tmin | | | deg. C | 0 | 6 | | 0 | 0 | 5 |
| Optimum temperature for growth | Topt | | | deg. C | 14 | 20 | | 12 | 15 | 27 |
| Maximum temperature for growth | Tmax | | | deg. C | 32 | 35 | | 30 | 40 | 35 |
| *Frost modifier (fFRost)* |  | | |  |  |  | |  |  |  |
| Days production lost per frost day | kF | | | days | 0.25 | 1.00 | | 1.00 | 0.50 | 3.00 |
| *Soil water modifier (fSW)* |  | | |  |  |  | |  |  |  |
| Moisture ratio deficit for f_q_ = 0.5 | SWconst | | | - | 0.7 | 0.7 | | 0.7 | 0.7 | 0.55 |
| Power of moisture ratio deficit | SWpower | | | - | 9 | 9 | | 9 | 9 | 6 |
| *Atmospheric CO2 modifier (fCO2)* |  | | |  |  |  | |  |  |  |
| Assimilation enhancement factor at 700 ppm | fCalpha700 | | | - | 1.4 | 1.4 | | 1.4 | 1.4 | 1.4 |
| Canopy conductance enhancement factor at 700 ppm | fCg700 | | | - | 0.7 | 0.7 | | 0.7 | 0.7 | 0.7 |
| *Fertility effects* |  | | |  |  |  | |  |  |  |
| Value of 'm' when FR = 0 | m0 | | | - | 0 | 0 | | 0 | 0 | 0 |
| Value of 'fNutr' when FR = 0 | fN0 | | | - | 0.6 | 0.6 | | 0.5 | 0.8 | 0.6 |
| Power of (1-FR) in 'fNutr' | fNn | | | - | 1 | 1 | | 1 | 1 | 1 |
| *Age modifier (fAge)* |  | | |  |  |  | |  |  |  |
| Maximum stand age used in age modifier | MaxAge | | | years | 70 | 60 | | 1000 | 60 | 400 |
| Power of relative age in function for fAge | nAge | | | - | 4 | 4 | | 4 | 4 | 4 |
| Relative age to give fAge = 0.5 | rAge | | | - | 0.50 | 0.95 | | 0.95 | 0.95 | 0.95 |

| **Meaning/comments** | **Name** | **Units** | | ***P. radiata*** | | ***E. fastigata*** | | ***S. sempervirens*** | | ***Ps. menziesii*** | | ***P. totara*** | |
| --- | --- | --- | --- | --- | --- | --- | --- | --- | --- | --- | --- | --- | --- |
| **Stem mortality & self-thinning** |  | |  | |  | |  | |  | |  | |  |
| Mortality rate for large t | gammaNx | | %/year | | 0.0 | | 5.5 | | 0.0 | | 0.0 | | 0.0 |
| Seedling mortality rate (t = 0) | gammaN0 | | %/year | | 0 | | 0 | | 0 | | 0 | | 0 |
| Age at which mortality rate has median value | tgammaN | | years | | 0 | | 8 | | 0 | | 0 | | 0 |
| Shape of mortality response | ngammaN | | - | | 1.0 | | 0.5 | | 0.5 | | 1.0 | | 1.0 |
| Max. stem mass per tree @ 1000 trees/hectare | wSx1000 | | kg/tree | | 200 | | 333 | | 370 | | 440 | | 900 |
| Power in self-thinning rule | thinPower | | - | | 1.5 | | 4.0 | | 2.5 | | 1.5 | | 1.2 |
| Fraction mean single-tree foliage biomass lost per dead tree | mF | | - | | 0 | | 0 | | 0 | | 0 | | 0 |
| Fraction mean single-tree root biomass lost per dead tree | mR | | - | | 0.2 | | 0.2 | | 0.2 | | 0.2 | | 0.2 |
| Fraction mean single-tree stem biomass lost per dead tree | mS | | - | | 0.2 | | 0.2 | | 0.2 | | 0.2 | | 0.2 |
| **Canopy structure and processes** |  | |  | |  | |  | |  | |  | |  |
| *Specific leaf area* |  | |  | |  | |  | |  | |  | |  |
| Specific leaf area at age 0 | SLA0 | | m^2^/kg | | 5 | | 11 | | 11 | | 11 | | 20 |
| Specific leaf area for mature leaves | SLA1 | | m^2^/kg | | 5 | | 7 | | 7 | | 4 | | 18 |
| Age at which specific leaf area = (SLA0+SLA1)/2 | tSLA | | years | | 2 | | 2.5 | | 2 | | 2.5 | | 2 |
| *Light interception* |  | |  | |  | |  | |  | |  | |  |
| Extinction coefficient for absorption of PAR by canopy | k | | - | | 0.5 | | 0.5 | | 0.5 | | 0.5 | | 0.5 |
| Age at canopy cover | fullCanAge | | years | | 3.000 | | 5.000 | | 3.000 | | 2.718 | | 15.000 |
| Maximum proportion of rainfall evaporated from canopy | MaxIntcptn | | - | | 0.15 | | 0.15 | | 0.15 | | 0.15 | | 0.15 |
| LAI for maximum rainfall interception | LAImaxIntcptn | | - | | 5 | | 3 | | 8 | | 10 | | 5 |
| **Meaning/comments** | **Name** | **Units** | | ***P. radiata*** | | ***E. fastigata*** | | ***S. sempervirens*** | | ***Ps. menziesii*** | | ***P. totara*** | |
| *Production and respiration* |  | |  | |  | |  | |  | |  | |  |
| Canopy quantum efficiency | alpha | | molC/molPAR | | 0.050 | | 0.060 | | 0.055 | | 0.060 | | 0.060 |
| Ratio NPP/GPP | Y | | - | | 0.47 | | 0.47 | | 0.47 | | 0.47 | | 0.47 |
| *Conductance* |  | |  | |  | |  | |  | |  | |  |
| Minimum canopy conductance | MinCond | | m/s | | 0 | | 0 | | 0 | | 0 | | 0 |
| Maximum canopy conductance | MaxCond | | m/s | | 0.02 | | 0.02 | | 0.03 | | 0.015 | | 0.02 |
| LAI for maximum canopy conductance | LAIgcx | | - | | 3.33 | | 3.33 | | 3.33 | | 5 | | 3.33 |
| Defines stomatal response to VPD | CoeffCond | | 1/mBar | | 0.05 | | 0.05 | | 0.05 | | 0.05 | | 0.05 |
| Canopy boundary layer conductance | BLcond | | m/s | | 0.2 | | 0.2 | | 0.2 | | 0.2 | | 0.2 |
| **Wood and stand properties** |  | |  | |  | |  | |  | |  | |  |
| *Branch and bark fraction (fracBB)* |  | |  | |  | |  | |  | |  | |  |
| Branch and bark fraction at age 0 | fracBB0 | | - | | 0.5 | | 0 | | 0.5 | | 0.5 | | 0.5 |
| Branch and bark fraction for mature stands | fracBB1 | | - | | 0.1 | | 0 | | 0.1 | | 0.1 | | 0.1 |
| Age at which fracBB = (fracBB0+fracBB1)/2 | tBB | | years | | 5 | | 0 | | 5 | | 5 | | 5 |
| *Basic Density* |  | |  | |  | |  | |  | |  | |  |
| Minimum basic density - for young trees | rhoMin | | t/m3 | | 0.480 | | 0.400 | | 0.480 | | 0.480 | | 0.480 |
| Maximum basic density - for older trees | rhoMax | | t/m3 | | 0.480 | | 0.500 | | 0.480 | | 0.480 | | 0.480 |
| Age at which rho = (rhoMin+rhoMax)/2 | tRho | | years | | 4 | | 10 | | 4 | | 4 | | 4 |
| *Stem height* |  | |  | |  | |  | |  | |  | |  |
| Constant in the stem height relationship | aH | | - | | 0.0000 | | 2.0800 | | 0.0000 | | 0.0000 | | 1.4093 |
| Power of DBH in the stem height relationship | nHB | | - | | 0.0000 | | 0.7000 | | 0.0000 | | 0.0000 | | 0.7345 |
| Power of stocking in the stem height relationship | nHN | | - | | 0.0000 | | -0.0036 | | 0.0000 | | 0.0000 | | 0.0000 |
| **Meaning/comments** | **Name** | | **Units** | | ***P. radiata*** | | ***E. fastigata*** | | ***S. sempervirens*** | | ***Ps. menziesii*** | | ***P. totara*** |
| Constant in the stem volume relationship | aV | | - | | 0.0000 | | 0.0000 | | 0.0000 | | 0.0000 | | 0.9796 |
| Power of DBH in the stem volume relationship | nVB | | - | | 0.0000 | | 0.0000 | | 0.0000 | | 0.0000 | | 1.859 |
| Power of stocking in the stem volume relationship | nVN | | - | | 0 | | 0 | | 0 | | 0 | | 0 |
| **Conversion factors** |  | |  | |  | |  | |  | |  | |  |
| Intercept of net v. solar radiation relationship | Qa | | W/m2 | | -90 | | -90 | | -90 | | -90 | | -90 |
| Slope of net v. solar radiation relationship | Qb | | - | | 0.8 | | 0.8 | | 0.8 | | 0.8 | | 0.8 |
| Molecular weight of dry matter | gDM_mol | | gDM/mol | | 24 | | 24 | | 24 | | 24 | | 24 |
| Conversion of solar radiation to PAR | molPAR_MJ | | mol/MJ | | 2.3 | | 2.3 | | 2.3 | | 2.3 | | 2.3 |

**Table S2. Ranking based on site (1 the best to 15 the worst), mean annual carbon sequestration (t/ha) and terms. CNI= Central North Island, NL= Northland and SL=Southland**

| **Species** | **Site** | | | | | | **Overall score** | |
| --- | --- | --- | --- | --- | --- | --- | --- | --- |
|  | **CNI** | | **NL** | | **SL** | |  |  |
|  | **Term** | | | | | |  |  |
|  | **Short** | **Long** | **Short** | **Long** | **Short** | **Long** | **Short** | **Long** |
| ***P. radiata*** | **1** | **1** | **1** | **3** | **2** | **1** | **4** | **5** |
| ***E. fastigata*** | **3** | **4** | **4** | **5** | **3** | **5** | **10** | **14** |
| ***P. menziesii*** | **2** | **3** | **3** | **4** | **1** | **2** | **6** | **9** |
| ***S. sempervirens*** | **4** | **2** | **2** | **1** | **4** | **3** | **10** | **6** |
| ***P. totara*** | **5** | **4** | **5** | **3** | **5** | **4** | **15** | **11** |
